# Delayed adaptive immunity is related to higher MMR vaccine-induced antibody titers in children

**DOI:** 10.1038/cti.2016.20

**Published:** 2016-04-29

**Authors:** Anna Strömbeck, Anna-Carin Lundell, Inger Nordström, Kerstin Andersson, Ingegerd Adlerberth, Agnes E Wold, Anna Rudin

**Affiliations:** 1 Department of Rheumatology and Inflammation Research at the Institute of Medicine, The Sahlgrenska Academy, University of Gothenburg, Gothenburg, Sweden; 2 Department of Infectious Diseases at the Institute of Biomedicine, The Sahlgrenska Academy, University of Gothenburg, Gothenburg, Sweden

## Abstract

There are notable inter-individual variations in vaccine-specific antibody responses in vaccinated children. The aim of our study was to investigate whether early-life environmental factors and adaptive immune maturation prior and close to measles–mumps–rubella (MMR) immunization relate to magnitudes of vaccine-specific antibody titers. In the FARMFLORA birth cohort, including both farming and non-farming families, children were immunized with the MMR vaccine at 18 months of age. MMR vaccine-induced antibody titers were measured in plasma samples obtained at 36 months of age. Infants' blood samples obtained at birth, 3–5 days and at 4 and 18 months of age were analyzed for T- and B-cell numbers, proportions of naive and memory T and B cells, and fractions of putative regulatory T cells. Multivariate factor analyses show that higher anti-MMR antibody titers were associated with a lower degree of adaptive immune maturation, that is, lower proportions of memory T cells and a lower capacity of mononuclear cells to produce cytokines, but with higher proportions of putative regulatory T cells. Further, children born by cesarean section (CS) had significantly higher anti-measles titers than vaginally-born children; and CS was found to be associated with delayed adaptive immunity. Also, girls presented with significantly higher anti-mumps and anti-rubella antibody levels than boys at 36 months of age. These results indicate that delayed adaptive immune maturation before and in close proximity to immunization seems to be advantageous for the ability of children to respond with higher anti-MMR antibody levels after vaccination.

Vaccines have a critical role for the protection against infectious diseases by inducing specific neutralizing IgG antibodies and immunological memory. In Sweden, measles–mumps–rubella (MMR) vaccination is routinely initiated at 18 months with a booster dose at 6–8 years of age, and 95% of all Swedish 2-year-olds have received the vaccine. There is however a notable inter-individual variation in magnitudes of vaccine-specific antibody titers in vaccinated children. Whether these variations are related to the maturation of the adaptive immune system before and close to vaccination is unknown. The adaptive immunity of newborns is essentially naive, given limited exposure to exogenous antigens *in utero*. Indeed, the majority of CD4^+^ T cells and B cells in cord blood are naive, that is, express the surface markers CD45RA and CD5, respectively.^1, 2, 3, 4, 5^ Along with an age-dependent decrease in naive lymphocytes there is an increase in proportions of memory T and B cells that express CD45RO and CD27, respectively.^1, 3, 4, 5^ This immune maturation progress is paralleled by an enhanced capacity to produce cytokines and with increased total IgG levels in blood.^4, 6, 7, 8, 9^ Regulatory T cells (Tregs), which express FOXP3 and/or CTLA-4, impede proliferation and cytokine production of other T cells,^10, 11, 12, 13^ but also have suppressive effects on dendritic cells as well as on B cells and immunoglobulin responses,^13, 14, 15^ and reviewed in Shevach.^16^ Whether higher proportions of Tregs in blood hamper the subsequent MMR vaccine-induced antibody titers in children is not known.

Early-life environmental factors have been shown to influence adaptive immune maturation. Growing up on a farm is associated with higher proportions of memory T cells and with an enhanced capacity to produce proinflammatory and T helper-associated cytokines.^17, 18^ Contrary, children born by cesarean section (CS) have lower proportions of memory T cells in childhood.^19^ Additionally, we have shown that boys have lower B-cell activating factor levels in cord blood than girls, as well as higher proportions of naive B cells at birth and later in childhood.^20^ Sex-related differences in vaccine-specific antibody levels have also been reported, demonstrating that girls present with higher mumps and rubella antibody titers.^21, 22^ Given that certain early-life environmental factors appear to have profound effects on immune maturation, they may also impact inter-individual variations in vaccine-induced antibody responses observed in children. By studying the FARMFLORA birth cohort, we sought to further explore how environmental factors and postnatal adaptive immune maturation relate to the MMR-specific humoral immune response.

## RESULTS

### Post-vaccination MMR antibody titers at 36 months of age

**Figure 1:**
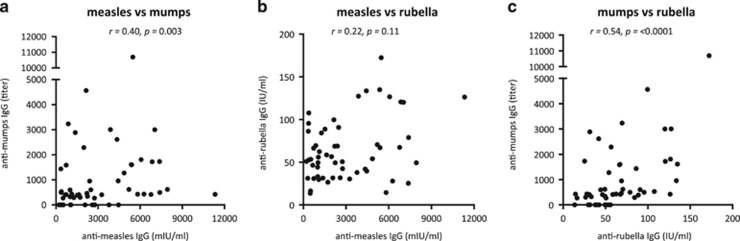
Correlation plots depicting post-vaccination plasma anti-measles IgG titers in relation to (**a**) anti-mumps IgG titers and (**b**) anti-rubella IgG titers, and (**c**) anti-rubella IgG titers in relation to anti-mumps IgG titers at 36 months of age (Spearman's rank correlation test). A *P*-value of ⩽0.05 was regarded as being statistically significant.

In the present study, MMR vaccine-induced antibody titers were measured in plasma samples obtained at 36 months of age, that is, approximately 18 months after the immunization. We first examined whether the respective vaccine-induced antibody titers correlated. We found a moderate correlation between anti-measles IgG levels (median 2201, range 217–11 328 mIU ml^−1^) and anti-mumps titers (median IgG titer 431, range 0–10 697), while anti-measles levels did not correlate to anti-rubella IgG levels (median 54, range 14–172 IU ml^−1^) (Figures 1a and b). There was also a moderate correlation between levels of anti-mumps and anti-rubella IgG (Figure 1c). At 36 months of age, all children presented with post-vaccination anti-measles and anti-rubella IgG levels above 120 mIU ml^−1^ and 10 IU ml^−1^, respectively. However, 13 children (24%) presented with anti-mumps IgG levels below the detection. In line with this, approximately 20% of all children who receive the mumps vaccine in Sweden are seronegative for vaccine-specific antibodies post-vaccination. Thus, children who respond with higher specific IgG levels to one of the live-attenuated viruses do not necessarily respond with higher levels to the other two included in the MMR vaccine.

### Higher anti-measles titers are associated with CS and delayed immune maturation

**Figure 2:**
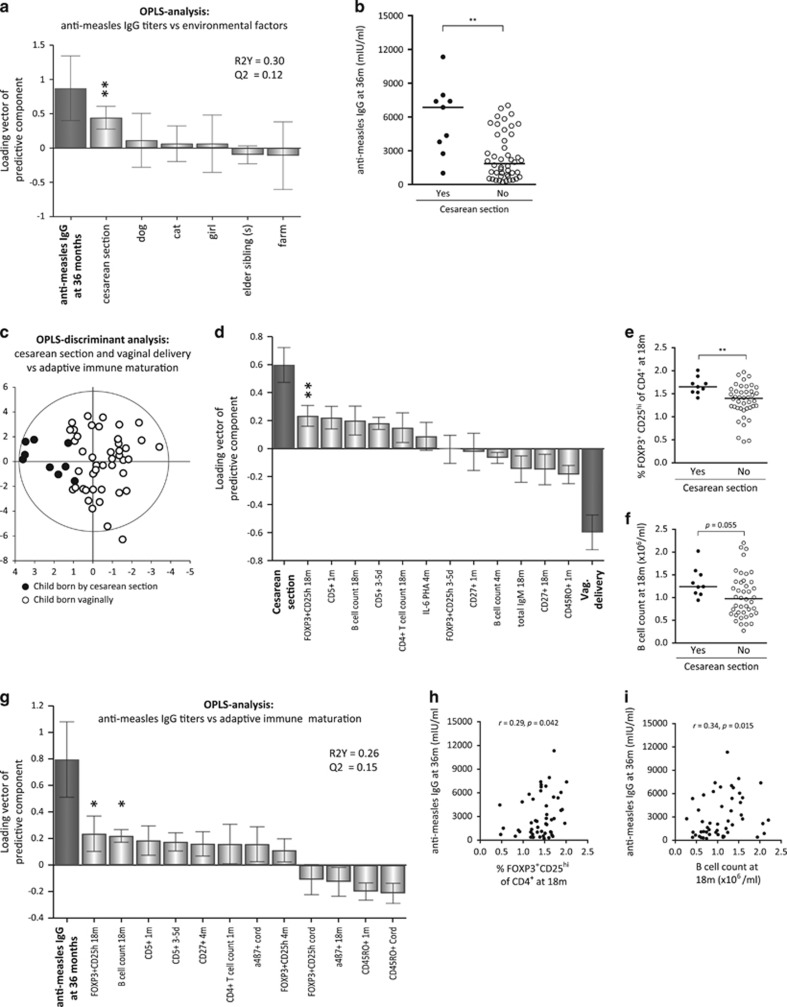
(**a**) OPLS plot depicting associations between Y, i.e., post-vaccination plasma levels of anti-measles IgG levels and X-variables, i.e., environmental factors. X-variables with bars projected in the same direction as the Y-variable are positively associated, whereas parameters in the opposite direction are inversely related to the Y-variable. The larger the bar and smaller the error bar, the stronger and more certain is the contribution to the model. The quality of the OPLS analysis is based on the parameters R2, i.e., how well the variation of the variables is explained by the model, and Q2, i.e., how well a variable can be predicted by the model. (**b**) Anti-measles IgG levels in CS or vaginally delivered children. (**c**) OPLS-DA observation plot separating CS and vaginally-born children based on immune maturation and the OPLS-DA loadings column plot in (**d**) shows the immune variables most associated with respective delivery mode. (**e**) Treg proportions and (**f**) B-cell numbers in relation to delivery mode. (**g**) OPLS-DA displaying associations between anti-measles IgG levels and immune maturation. (**h**, **i**) Correlations between Treg proportions or B-cell numbers and anti-measles IgG levels. A *P*-value of ⩽0.05 was regarded as being statistically significant (*P⩽0.05 and **P⩽0.01), Mann–Whitney *U*-test (**b**, **e**, **f**) or Spearman's correlation test (**h**–**i**).

As vaccine-induced immune responses may be influenced by demographic variables, we examined whether higher anti-measles antibody titers were associated with growing up on a dairy farm, having elder sibling(s), having pets, delivery mode or with the sex of the child. By multivariate factor analysis (Figure 2a), we show that higher anti-measles vaccine-induced IgG levels (Y-variable to the far left) were associated with delivery by CS, as illustrated by a bar pointing in the same direction as that representing high vaccine-induced anti-measles titers. Univariate analysis confirmed that children born by CS had significantly higher anti-measles IgG titers at 36 months of age than vaginally delivered children (Figure 2b). Even though a relatively small number of children were delivered by CS, all except one had anti-measles IgG levels above the median value for those who were delivered vaginally. All other factors assessed were unrelated to magnitudes of anti-measles titers in these children (Figure 2a).

We next analyzed the relationship between delivery mode and the early maturation of the adaptive immune system. X-variables representing adaptive immune maturation included flow-cytometry data regarding numbers of CD4^+^ T cells and B cells, proportions of CD4^+^ T cells that were FOXP3^+^CD25^high^ or CTLA-4^+^CD25^+^, as well as T- and B-cell subsets at different maturational stages, for example, proportions of CD45RO^+^ T cells, CD5^+^ or CD27^+^ B cells at 3–5 days, 1, 4 and 18 months of age, phytohemagglutinin (PHA)-induced cytokine production by mononuclear cells at 4 and 18 months and total immunoglobulin levels at 18 months of age. The orthogonal projections to latent structure discriminant analysis (OPLS-DA) score scatter plot in Figure 2c indicated that children delivered by CS or vaginally could be separated based on the included immune parameters. Parameters that displayed the strongest association, positive or negative, with the respective delivery mode are identified in the OPLS-DA loadings column plot in Figure 2d. CS was associated with higher proportions of FOXP3^+^CD25^high^ T cells, higher B-cell counts, as well as with higher proportions of CD5^+^ immature/naive B cells (Figures 2d–f). In contrast, vaginal delivery was associated with higher proportions of infantile CD45RO^+^ memory T cells and with CD27^+^ memory B cells. The gating strategy for the various T- and B-cell subsets is presented in Supplementary Figure S1, and PHA-induced cytokine levels produced by mononuclear cells at 4 and 18 months of age have been published previously.^17^ In conclusion, even though only nine children were delivered by CS in the present cohort our results indicate that CS is associated with a delayed adaptive immune maturation, which is in line with a previous study demonstrating lower proportions of memory CD4^+^ T cells among CS-delivered children.^19^

It is not known whether magnitudes of MMR vaccine-induced titers are related to the maturation progress of the adaptive immune system before and in close proximity to vaccination. The OPLS analysis in Figure 2g shows that higher anti-measles IgG levels (Y-variable located to the far left) were most strongly associated with higher proportions of FOXP3^+^CD25^high^ T cells and higher B-cell numbers at 18 months of age, as well as with higher proportions of CD5^+^ immature/naive B cells but with lower proportions of CD45RO^+^ memory T cells in early infancy (Figure 2g). Multivariate associations were followed up by univariate analyses and significant correlations are shown in Figures 2h and i. Together, these results indicate that CS-delivered children responded significantly stronger to measles vaccine and suggest that one mechanism might be the delayed adaptive immune maturation caused by CS.

### Higher anti-mumps and anti-rubella titers are related to delayed T-cell maturation

**Figure 3:**
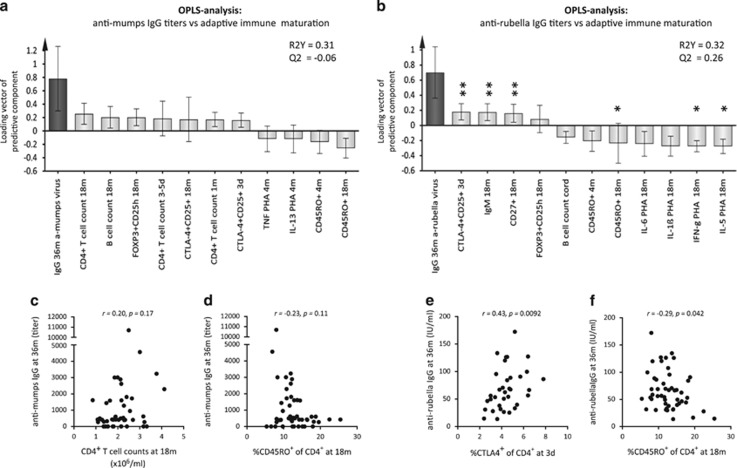
OPLS loadings column plots depicting associations between Y, i.e., post-vaccination plasma (**a**) anti-mumps and (**b**) anti-rubella IgG titers and X-variables, i.e., adaptive immune maturation. X-variables with bars projected in the same direction as the Y-variable are positively associated, whereas parameters in the opposite direction are inversely related to the Y-variable. The larger the bar and smaller the error bar, the stronger and more certain is the contribution to the model. The quality of the OPLS analysis is based on the parameters R2, i.e., how well the variation of the variables is explained by the model, and Q2, i.e., how well a variable can be predicted by the model. Correlations between (**c**) CD4^+^ T-cell numbers or (**d**) CD45RO^+^ T cells and anti-mumps IgG titers. Correlations between (**e**) CTLA-4^+^CD4^+^ T cells or (**f**) CD45RO^+^ T cells and anti-rubella IgG titers. A *P*-value of ⩽0.05 was regarded as being statistically significant (*P⩽0.05 and **P⩽0.01), Spearman's correlation test (**c**–**f**).

Next, we investigated associations between higher vaccine-induced anti-mumps and anti-rubella IgG titers (Y-variables located to the far left) and adaptive immune maturation (X-variables) by the use of multivariate factor analyses. The most apparent pattern displayed for both anti-mumps and anti-rubella IgG titers was that higher titers were associated with lower proportions of CD45RO^+^ memory T cells as well as with lower PHA-induced cytokine production (Figures 3a and b). Additionally, higher anti-mumps titers were positively associated with higher numbers of CD4^+^ T cells and B cells as well as with higher proportions of putative Tregs (Figure 3a). Higher anti-rubella IgG titers were positively associated with higher proportions of putative Tregs, but were also related to higher total IgM levels and to higher proportions of CD27^+^ memory B cells (Figure 3b). Multivariate associations were followed up by univariate analyses. No statistically significant correlations were found between anti-mumps IgG levels and the immune variables that displayed the strongest association (Figures 3c and d). For anti-rubella IgG levels, on the other hand, the proportions of CTLA-4^+^ T cells correlated positively and the proportions of CD45RO^+^ T cells correlated inversely to the vaccine-induced antibody levels (Figures 3e and f). All statistically significant correlations are indicated with asterisks in Figure 3b. These results indicate that higher anti-mumps and anti-rubella IgG titers are related to delayed T-cell maturation, which was also observed for higher anti-measles IgG titers.

### Girls present with higher anti-mumps and anti-rubella titers than boys

**Figure 4:**
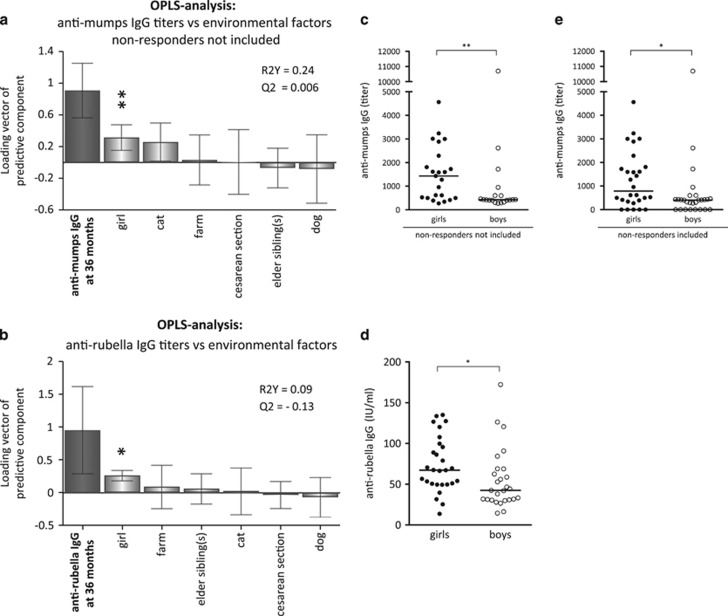
OPLS plot depicting associations between Y, i.e., post-vaccination (**a**) anti-mumps and (**b**) anti-rubella IgG titers and X-variables, i.e., environmental factors. X-variables with bars projected in the same direction as the Y-variable are positively associated, whereas parameters in the opposite direction are inversely related to the Y-variable. The larger the bar and smaller the error bar, the stronger and more certain is the contribution to the model. The quality of the OPLS analysis is based on the parameters R2, i.e., how well the variation of the variables is explained by the model, and Q2, i.e., how well a variable can be predicted by the model. (**c**) Anti-mumps (non-responders not included), (**e**) anti-mumps (non-responders included) and (**d**) anti-rubella IgG levels in girls and boys. A *P*-value of ⩽0.05 was regarded as being statistically significant (*P⩽0.05 and **P⩽0.01), Mann–Whitney *U-*test (**c**–**e**).

When magnitudes of anti-mumps and anti-rubella IgG titers (Y-variables located to the far left) were assessed in relation to demographic factors (X-variables), we found that female sex, but not the other factors studied, was associated with higher vaccine-induced titers (Figures 4a and b). Indeed, girls demonstrated with significantly higher anti-mumps and rubella IgG titers than boys (Figures 4c and d). It should be noted that children who were seronegative for vaccine-specific anti-mumps antibodies were not included in the OPLS analyses as they cannot be related to variables classified into yes or no. However, girls presented with significantly higher anti-mumps IgG titers than boys also when non-responders were included in the univariate analysis (Figure 4e).

### Postnatal immune maturation is more rapid in girls

**Figure 5:**
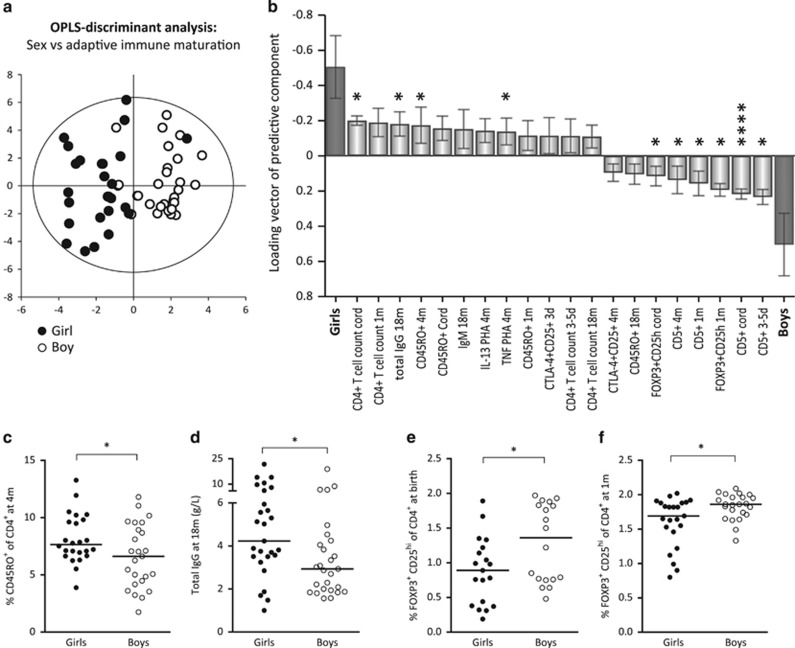
(**a**) OPLS-DA observation plot separating girls and boys based on immune maturation and (**b**) shows immune variables most associated with respective sex. (**c–f**) Sex differences in total IgG, CD45RO^+^ T cells and FOXP3^+^CD25^high^CD4^+^ T cells. A *P*-value of ⩽0.05 was regarded as being statistically significant (*P⩽0.05 and ****P⩽0.0001), Mann–Whitney *U*-test (**c**–**f**).

As higher anti-mumps and anti-rubella titers were associated with delayed T-cell immunity (Figure 3), we examined whether adaptive immune maturation was different in girls and boys. The OPLS-DA score scatter plot indicated that girls and boys could be separated based on the included immune parameters representing adaptive immune maturation described above (Figure 5a). Female sex was associated with higher numbers of CD4^+^ T cells, higher proportions of CD45RO^+^ memory T cells, higher levels of total IgG and IgM as well as with higher PHA-induced cytokine production (Figures 5b–d). Male sex was primarily associated with higher proportions CD5^+^ immature/naive B cells, as previously shown for the whole FARMFLORA study (*n*=65),^20^ as well as with higher proportions of putative Tregs (Figures 5b). Thus, the adaptive immune maturation differs significantly between girls and boys during the first 18 months of life, which may contribute to the sex-linked differences observed in vaccine-induced anti-mumps and anti-rubella IgG responses.

## DISCUSSION

In this study, we demonstrate how certain environmental factors and postnatal adaptive immune maturation before MMR immunization relate to the subsequent antibody response. One principal finding was that higher anti-MMR antibody titers were associated with delayed T-cell maturation. Another finding was that children born by CS presented with significantly higher vaccine-induced anti-measles IgG levels than vaginally-born children, and CS was found to be related to delayed adaptive immunity.

Even though the rate of CS deliveries has increased rapidly in both US and Europe over the last decades,^23, 24^ there is very limited knowledge regarding its effect on postnatal immune maturation as well as on vaccine-induced antibody responses in children. For the first time, we here demonstrate that children born by CS had significantly higher anti-measles titers at 36 months of age than children born vaginally. Moreover, multivariate factor analysis demonstrated a clear distinction in adaptive immune maturation over the first 18 months of life based on delivery mode. CS was associated with higher proportions of putative Tregs, naive B cells and higher numbers of lymphocytes, while vaginal delivery was related to higher proportions of memory T and B cells. Even though only 9 children were delivered by CS in the present cohort, our results are in line with a recent study demonstrating that 2–10 years old CS-delivered children present with lower proportions of memory T cells compared to children delivered vaginally.^19^ Furthermore, the multivariate association pattern found between CS and immune maturation was mirrored in the analysis describing the relationship between higher anti-measles titers and immune maturation for the entire cohort. Thus, the present study indicates that delivery by CS is linked to delayed adaptive immune maturation, which seems to be beneficial for higher anti-measles titers in children.

In contrast to our results regarding CS and higher vaccine-induced anti-measles titers, we recently showed that delivery mode was unrelated to post-vaccination anti-DTP antibody levels.^25^ Similarly, CS has been shown to be unrelated to vaccine-induced tetanus antibody titers in serum over the first 2 years of life.^19^ However, a direct comparison between CS in relation to antibody responses to bacterial toxin/toxoid-based vaccines with that of antibody responses to live-attenuated virus-based vaccines might however be somewhat misleading as the vaccines differ in composition and are administered at different ages. Owing to these differences combined with the fact that only nine children were delivered by CS in the present study, additional studies in other cohorts are required to confirm the relationship between CS, delayed postnatal immune maturation and higher vaccine-induced anti-measles antibody response in children.

In the present study, delayed adaptive immune maturation was not only related to higher anti-measles, but also to higher anti-mumps and anti-rubella titers. Indeed, anti-mumps and anti-rubella titers were negatively associated with the proportions of memory T cells as well as with a decreased capacity of mononuclear cells to produce cytokines before and in close proximity to immunization. On the contrary, higher antibody titers were associated with higher proportions of putative Tregs, which has previously been linked to delayed T-cell maturation.^4^ In line with our results, young healthy adults in Switzerland had higher yellow fever vaccine (YF17D)-induced titers compared to vaccinees in Uganda, and the former group presented with lower proportions of memory T-cell subsets and higher fractions of naive B cells before vaccination.^26^ Moreover, viral-based oral vaccines, for example, bovine rotavirus and polioviruses, are more immunogenic in industrialized than in developing countries,^27, 28, 29^ but the reasons for these differences are still poorly understood. Together, these results imply that a more immature adaptive immunity may favor higher antibody levels to live-attenuated virus vaccines. Thus, these results may be generalizable for at least some attenuated virus vaccines.

In contrast to the present results regarding virus vaccines and immune maturation, it has been shown that Turkish infants respond with higher diphtheria-tetanus-pertussis (DTP) vaccine-specific antibody levels compared with Belgian infants.^30^ Further, Senegalese infants who received the same batch of DTP vaccine presented with post-vaccination titers in the same range as the Turkish infants.^30, 31^ In line with this, we recently showed that earlier infantile immune maturation is related to higher DTP vaccine responses in children.^25^ Thus, in view of results obtained from our cohort and those from other studies discussed above it appears as some vaccines might benefit from a more immature/naive immune system, while others are more effective if the immune system is more mature/activated.

Although we and others have demonstrated that exposure to a farming environment affects the maturation of the immune system, both pre- and postnatally,^17, 18, 20^ MMR titers did not differ between farmers' and non-farmers' children in the present study. Possibly, the divergence in immune maturation between these two groups of children, living in the same rural area, might not be distinct enough to affect the MMR antibody responses. Male sex was, however, associated with delayed adaptive immune maturation. We recently demonstrated that boys have higher proportions of naive CD5^+^ B cells in blood over the first 3 years of life than girls.^20^ Additionally, we here show that boys have higher proportions of putative Tregs, lower fractions of memory T cells and mononuclear cells with a lower capacity to produce cytokines in infancy. Even though a more immature immunity was advantageous for higher antibody titers to all three viruses in the MMR vaccine, boys presented with lower levels of both anti-mumps and rubella titers than girls. No difference in anti-measles titers was, however, observed between the sexes. Similar observations have previously been described in young adolescents.^21, 22, 32^

A limitation of this study is that only blood T- and B-cell subsets were examined, which may not be the most appropriate organ for this analysis. The relatively small size of this study may also be a limitation, but the study was still large enough to generate statistically significant results and permitted detailed follow-up regarding immunological analyses and demographic documentation. Advantages of our study are that it is prospective in nature and that multivariate factor analysis was employed to investigate how environmental factors and postnatal adaptive immune maturation before MMR immunization relate to the subsequent antibody responses.

The main finding from the current study is the association between delayed adaptive immune maturation and higher magnitudes of MMR vaccine-induced antibody titers. We also show that children born by CS had increased anti-measles titers and were associated with a more immature adaptive immune system, which could thus be a possible mechanism for higher anti-measles titers. A better understanding of the relationship between early-life environmental factors, immune maturation and vaccine responsiveness may lead to novel vaccination strategies.

## METHODS

### Subjects

In total, 55 Swedish infants from the prospective FARMFLORA study (*n*=65) whose parents agreed to take part in this part of the study were included.^17^ Among these children, 28 (51%) were girls, 9 (16%) were delivered by CS and 25 (45%) were raised on small dairy farms while 30 children lived on the country-side in the same area but not on farms. Blood samples were obtained from the umbilical cord, and peripheral blood was sampled at 3–5 days, and at 1, 4, 18 and 36 months of age. The children received MMR vaccine (M-M-R II, Merck, Kenliworth, NJ, USA) at approximately 18 months of age. All parents provided written informed consent for their children, and the study was approved by the Human Research Ethics Committee of the Medical Faculty, University of Gothenburg, Sweden.

### Quantification of antibodies in plasma

Concentrations of IgG antibodies against measles, mumps and rubella virus at 36 months of age were measured by Enzygnost IgG ELISA kit assays, according to the manufacturer's instructions (Siemens Healthcare Diagnostics, Marburg, Germany). Specific anti-measles and anti-rubella antibody titers 120–200 mIU ml^−1^ and 10–15 IU ml^−1^, respectively, correlate with protection (reviewed in Thakur *et al.*^33^). Anti-mumps antibody levels that correlate with protection are not defined.^33^ Total IgM, IgA and IgG levels in plasma at 18 months of age were determined by in-house ELISA as previously described in detail.^20^ All antibodies and standards were purchased from Jackson ImmunoResearch (Suffolk, England) and Calbiochem (Darmstadt, Germany).

### Measurement of cytokine production

Mononuclear cells isolated from peripheral blood samples obtained at 4 and 18 months of age were stimulated with 5 μg ml^−1^ PHA for 24 h, as previously described.^17^ Concentrations of IL-1β, IL-6, TNF, IFN-γ, IL-5 and IL-13 were measured in the supernatant by Flow Cytomix (eBioscience, Vienna, Austria).

### Flow cytometry

Total numbers and phenotypic characterization of T and B cells in the FARMFLORA study were determined by flow cytometry as previously described in detail.^4, 5^ For quality assurance, the same buffers, fluorochromes and antibody clones were used for analysis of cells in umbilical cord blood as well as in peripheral blood sampled at 3–5 days, and at 1, 4 and 18 months of age. The following antihuman monoclonal antibodies from BD Biosciences (Erembodegen, Belgium) were used: PerCP-conjugated anti-CD4 (clone SK3) and anti-CD20 (clone L27); allophycocyanin (APC)-conjugated anti-CD25 (clone 2A3) and anti-CD5 (clone UCHT2); phycoerythrin (PE)-conjugated anti-CD45RO (clone UCHL-1); FITC-conjugated anti-CD27 (clone L128) and biotin-conjugated anti-CTLA-4 (clone BN13). The PE-antihuman FOXP3-staining set (clone PCH101) was purchased from eBiosciences (San Diego, CA, USA); and the Cytofix/Cytoperm kit (BD Biosciences) was used for detection of CTLA-4. Samples were run in an FACS-Calibur (BD Biosciences) equipped with CellQuestPro software. All samples were analyzed with FlowJo software (TreeStar, Ashland, OR, USA). Gating strategies for T- and B-cell subsets are demonstrated in Supplementary Figure S1.

### Statistical analysis

*Multivariate factor analysis* (SIMCA Software, Umetrics, Umeå, Sweden): OPLS was implemented to investigate associations between a selected Y-variable and X-variable in linear multivariate models. OPLS-DA is a maximum separation projection that relies on X-variables and is guided by class information (Y-variables), for example, delivery mode and sex. VIP (variable importance for projection) values were used to identify X-variables that associate most strongly with the respective Y-variables. A representative full VIP plot for Figure 2g, which includes all immune parameters assessed and their contribution to the OPLS model, is shown in Supplementary Figure S2.

In the OPLS analyses, the importance of each X-variable to the Y-variable is represented by bars. The larger the bar and smaller the error bar, the stronger and more certain is the contribution to the model. The scale presented on the Y-axis of the OPLS plot is a dimensionless scale, the loading vector is normalized to length one. The quality of OPLS analyses is based on R2, how well the variation of the variables is explained by the model, and Q2, how well a variable can be predicted. Univariate analyses were performed exclusively on the X-variables that contributed most to the respective multivariate model to avoid biased preselection of early-life environmental factors and immune maturation variables and to avoid mass significance. *Univariate analyses* (GraphPad Software, La Jolla, CA, USA): Spearman's rank correlation test (Figures 1a–c, 2h–i and 3c–f) or two-tailed Mann–Whitney *U*-test (Figures 2b,e,f, 4c,d and g–j) was performed on X-variables that contributed most to the respective OPLS models to avoid mass significance. Statistically significant correlations or differences are indicated with asterisks in the respective OPLS plots; *P⩽0.05, **P⩽0.01 and ****P⩽0.0001.

## Figures and Tables

**Table 1:** Antibodies used for characterization of T and B cells

*Monoclonal antibodies*	*Fluorochrome*	*Clone*	*Company*
CD4	PerCP	SK3	BD Biosciences, Erembodegem, Belgium
CD25	APC	2A3	BD Biosciences
CD45RO	PE	UCHL-1	BD Biosciences
FOXP3	PE	PCH101	eBioscience, San Diego, CA, USA
biotin-CTLA-4	PE-streptavidin	BNI3	BD Biosciences
CD20	PerCP	L27	BD Biosciences
CD5	APC	UCHT2	BD Biosciences
CD27	FITC	L128	BD Biosciences
