# Supplementary material for: Delayed adaptive immunity is related to higher MMR vaccine-induced antibody titers in children
Source: Clin Transl Immunology. 2016 Apr 29;5(4):e75–. doi: 10.1038/cti.2016.20 (PMC4855269; doi:10.1038/cti.2016.20)
Supplement: Supplementary Information [file cti201620x1.pdf]

Figure S1

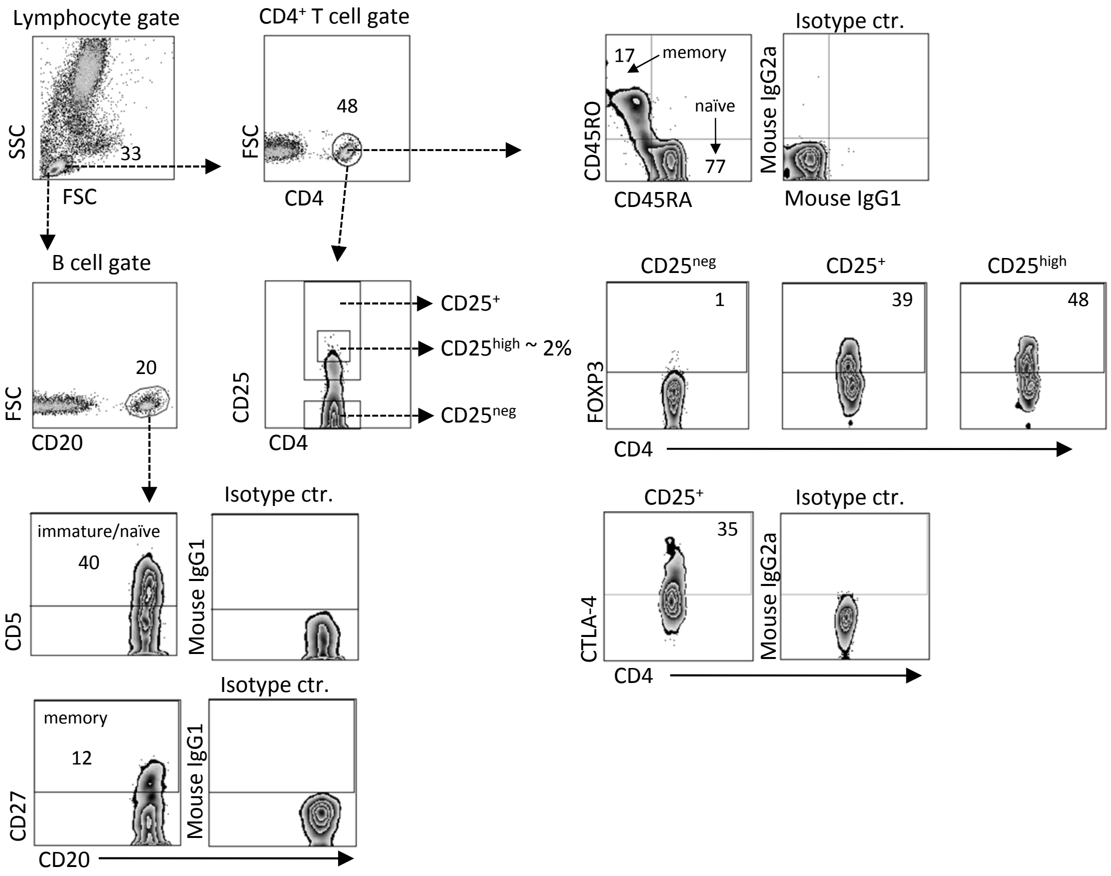

Figure S1. Representative flow cytometry plots showing our gating strategies in the FARMFLORA-study. Lymphocytes were defined in forward versus side scatter plot. T- and B cells were then gated according to their CD4 or CD20 expression, respectively. The CD45RO$^+$CD45RA$^-$ memory cells within the CD4$^+$ T cells population were gated based on the isotype control. CD4$^+$ T cells were also gated based on CD25^{neg}, CD25$^+$ or CD25^{high} expression. FOXP3$^+$CD25^{high} T cells were gated based on the lack of expression within the CD25^{neg} subset, and CTLA-4$^+$CD25$^+$ T cells were gated based on the isotype control. The CD5$^+$ immature/naive or CD27$^+$ memory B cell gate were based on respective isotype controls. Numbers represent the percentage of cells within the gate.

Figure S2

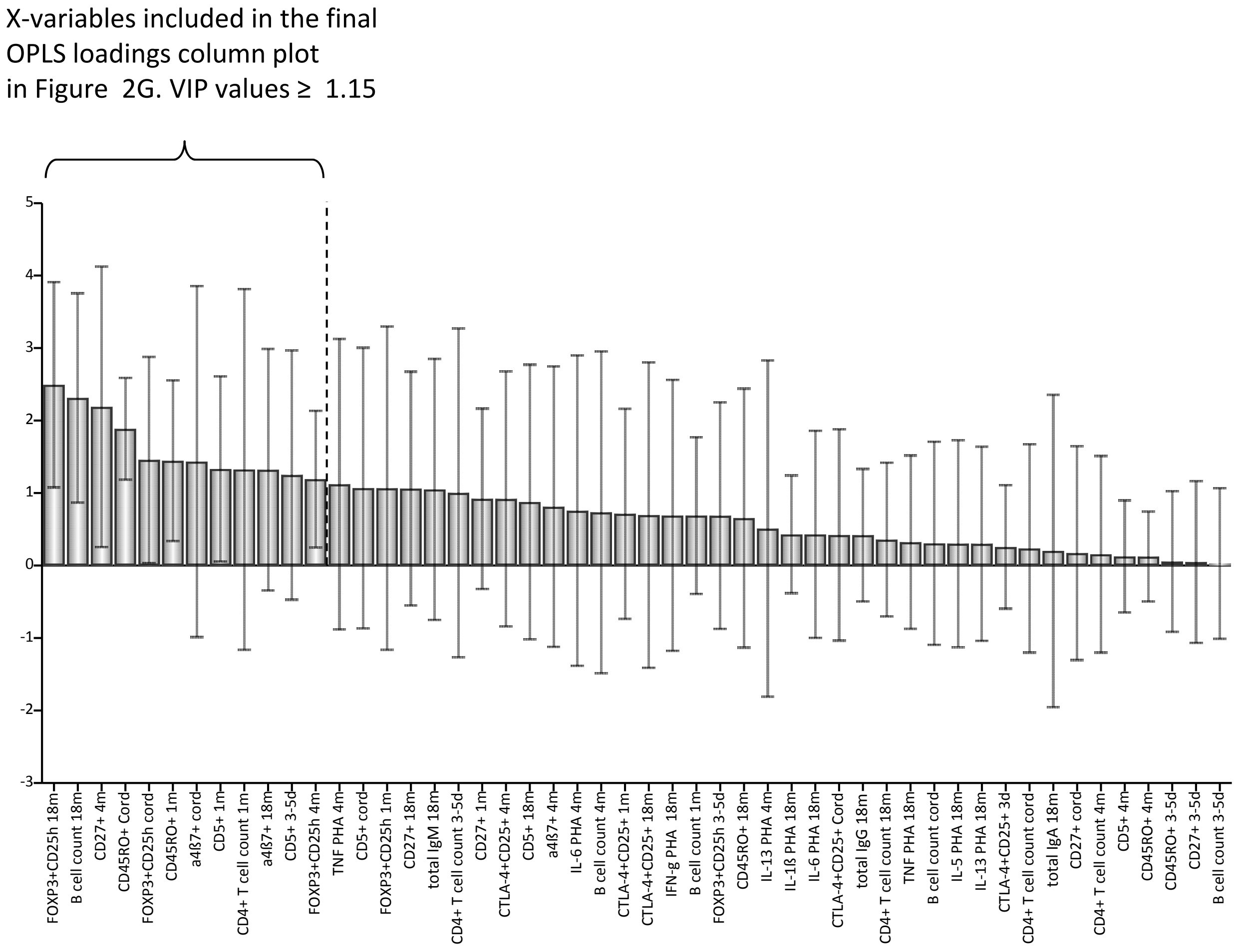

Figure S2. Representative variable importance for the projection (VIP) plot derived from the OPLS loadings column plot in figure 2G depicting associations between post-vaccination plasma titers of anti-measles IgG at 36 months of age (Y-variable) and T- and B cell variables that characterize adaptive immune maturation up to 18 months of age (X-variables). VIP summarize the importance of the X-variables both to explain X and to correlate to Y. The plot is sorted to display the largest VIP to the left. Confidence intervals are derived from jackknifing.
